# Subcellular Distribution of Thyroid Hormone Receptor Beta in Ovarian Cancer

**DOI:** 10.3390/ijms23052698

**Published:** 2022-02-28

**Authors:** Sabine Heublein, Udo Jeschke, Cornelia Sattler, Christina Kuhn, Anna Hester, Bastian Czogalla, Fabian Trillsch, Sven Mahner, Doris Mayr, Elisa Schmoeckel, Nina Ditsch

**Affiliations:** 1 Department of Obstetrics and Gynecology, University of Heidelberg, 69120 Heidelberg, Germany; cornelia.sattler@uksh.de; 2 Department of Obstetrics and Gynecology, University Hospital LMU Munich, 81377 Munich, Germany; udo.jeschke@med.uni-augsburg.de (U.J.); christina.kuhn@uk-augsburg.de (C.K.); anna.hester@med.uni-muenchen.de (A.H.); bastian.czogalla@med.uni-muenchen.de (B.C.); fabian.trillsch@med.uni-muenchen.de (F.T.); sven.mahner@med.uni-muenchen.de (S.M.); nina.ditsch@uk-augsburg.de (N.D.); 3 Department of Obstetrics and Gynecology, University of Augsburg, 86156 Augsburg, Germany; 4 Department of Pediatrics, University Medical Center Schleswig-Holstein, 24105 Kiel, Germany; 5 Department of Pathology, LMU Munich, 80337 Munich, Germany; doris.mayr@med.uni-muenchen.de (D.M.); elisa.schmoeckel@med.uni-muenchen.de (E.S.)

**Keywords:** ovarian cancer, thyroid hormone receptor beta, prognosis

## Abstract

Background: Since the most well-known function of thyroid hormone receptors (TRs) relies on their ability to act as ligand-activated transcription factors, their subcellular localization has been recognized to be relevant for their biological meaning. The current study aimed to determine the prevalence and subcellular distribution of TR beta and TR beta-1 in ovarian cancer (OC). Methods: Tissue was collected from 153 patients that had undergone surgery due to OC at the Department of Obstetrics and Gynaecology of the Ludwig-Maximilians-University Munich. Immunohistochemistry detecting TR beta and TR beta-1 was performed. Staining signals were quantified and tested for association with clinico-pathological parameters including overall survival (OS). Results: The subcellular distribution of TR beta and TR beta-1 differed among histologic subtypes, grade and FIGO stage. TR beta positivity was strongly linked to shortened overall survival (*p* < 0.001). Strikingly, this shortened OS was mainly attributed to those cases showing complete (*p* = 0.005) or incomplete shift of TR beta to the cytoplasm (*p* < 0.001). Significance was lost in multivariate testing. Conclusions: Cytoplasmatic localization of TR beta was associated with reduced OS, at least in univariate analysis. Since TRs have long been supposed to mainly function via the regulation of gene transcription in the nucleus, cytoplasmatic shifting might be interpreted as a regulator of their activity.

## 1. Introduction

Thyroid hormones are prominent regulators of cellular processes linked to differentiation, metabolism, apoptosis and growth [1,2]. Their most well-known mechanism of action depends on their ability to bind to thyroid hormone receptors (TRs). TRs build (hetero-) dimers and act as ligand-activated transcription factors on thyroid response elements located in the promotors of target genes [3]. So far, six different TR isoforms have been identified: TR alpha 1-3 and TR beta 1-3. Their expression is known to be tissue dependent, whereby TR alpha-1/2 and TR beta-1 seem to be the most relevant and widely expressed TRs [1].

Thyroid hormones and their receptors have been linked to carcinogenesis since the 1980s when TR alpha-1 was discovered to be the cellular counterpart of the retroviral oncogene v-erbA [4]. Moreover, somatic mutations in TRs have been identified to be present not only in thyroid cancer, but also in neoplasms deriving from breast, liver, kidney and pituitary gland [5]. Further evidence that TRs play an important role in tumor biology derives from in vivo and in vitro models. For instance, hepatoma growth was slowed down by the iatrogenic induction of hypothyroidism in rats. On the opposite treatment of different cell lines with thyroid hormones resulted in increased proliferation and angiogenesis [1,6]. The latter effect was effectively blocked by applying Tetrac, an antagonist of T3 [7].

For ovarian cancer, there is both epidemiologic and experimental evidence that links ovarian carcinogenesis to thyroid hormones and TRs. A population-based case–control study showed that the risk of ovarian cancer was almost doubled by the co-occurrence of thyroid dysfunction [8]. Second, experimental studies in primary ovarian surface epithelial (OSE) cells—the cell type that is hypothesized to give rise to ovarian cancer—demonstrated that TRs are strongly expressed on both the mRNA and protein levels [9,10]. Furthermore, the stimulation of OSE with thyroid hormone (T3) induced an inflammatory gene-expression profile and up-regulated estrogen receptor alpha and matrix metalloproteinase 9 [10]. We also recently demonstrated TR alpha to be expressed in ovarian cancer, and to predict prognosis [11]. Building on these results dealing with TR alpha, the current study aimed to investigate whether TR beta (as detected by an antibody not distinguishing TR beta subtypes), as well as its isoform TR beta-1 (as detected by an antibody specific for the beta-1 isoform), could also be identified in ovarian cancer tissue and might be associated with clinico-pathological parameters. There is increasing evidence that TRs, besides their ‘classical’ role as transcription factors, might also act via non-genomic mechanisms, e.g., by directly interacting with PI3K [12]. As such interactions are localized outside the nucleus in the cellular cytoplasm, the current study set a special focus on the subcellular distribution of TR beta/TR beta-1.

## 2. Results

### 2.1. Study Cohort

In total, 153 patients were evaluated (Table 1). Most tumors were of high-grade serous histology (*n* = 82; 54%). Low grade serous ovarian cancer was diagnosed in 26 cases (17%), while the remaining histology subtypes were endometrioid (14%), clear cell (8%) and mucinous (8%). About two thirds (109 out of 152) of the whole cohort were staged as either FIGO III or IV. Involvement of retroperitoneal lymph nodes and patient age were evenly distributed (see Table 1). The end point assessed was median overall survival, which was 3.3 years (95% CI: 2.1–4.5). Median follow up was 12.2 years (95% CI: 9.7–14.6). Due to the retrospective character of the study, our data, unfortunately, did not comprise the surgical resection status.

### 2.2. TR Beta/TR Beta-1 in Ovarian Cancer Tissue

**Figure 1:**
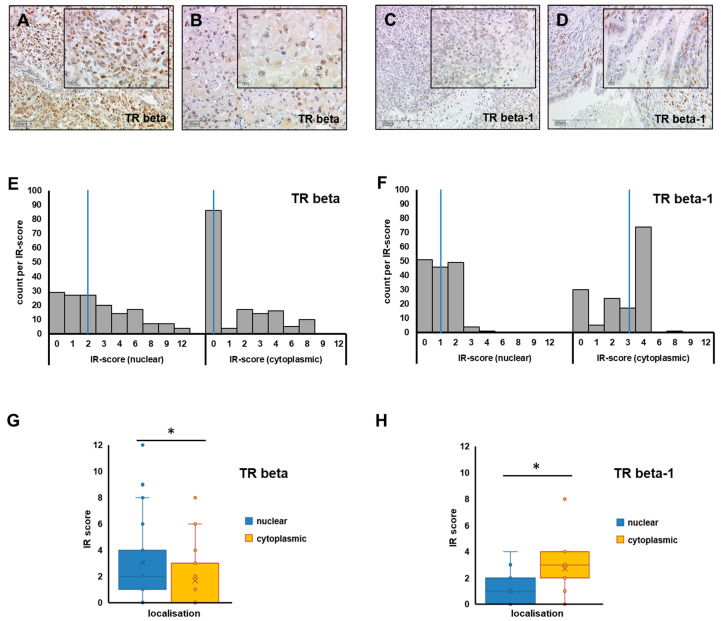
TR beta/TR beta-1 in ovarian cancer tissue. Representative images of TR beta (**A**,**B**) and TR beta-1 (**C**,**D**) as stained by immunohistochemistry in different OC histologic subtypes (A: high-grade serous, B: clear cell, C: endometroid, D: mucinous) are shown (**A**–**D**). Scale bars in images (**A**–**D**) represent 200 µm, and scale bars in insets are 100 µm. Nuclear and cytoplasmic staining was assessed independently and quantified by applying the IR-score. The number of cases per IR-score are plotted as histograms (**E**,**F**), and the median IR-score for each analysis (E: TR beta, F: TR beta-1) is highlighted by a blue vertical line, respectively. To compare median IR-scores of nuclear (blue) vs. cytoplasmic (yellow) staining, box plots (**G**: TR beta, **H**: TR beta-1) were plotted and differences were tested for statistical significance. Significant differences (*p* < 0.001), as determined by relevant Mann–Whitney U tests in G and H, are indicated by stars (*).

TR beta, as well as its isoform TR beta-1, was assessable in 151 and 152 cases, respectively (Figure 1A–D). Though TR beta and beta-1 are formally known as nuclear receptors, the locations of both TR beta and TR beta-1 were also observed in the cellular cytoplasm. Hence, nuclear and cytoplasmic signal were assessed independently. Regarding TR beta, a nuclear stain was detected with a median immune-reactive score (IRS) of 2 (range 0–12), while median IRS regarding cytoplasmic stain was significantly lower (median IRS = 0 (range 0–8), *p* < 0.001). A cytoplasmic staining signal was more common in the case of the beta-1 isoform (median IRS = 3, range 0–8), and was even more prominent than nuclear stain (median IRS = 1, range: 0–4, *p* < 0.001) (Figure 1E–H). Nuclear and cytoplasmic scores of TR beta and beta-1 were further tested for their correlation with estrogen (ER alpha and beta) and progesterone receptors (PRA and PRB). Nuclear TR beta was positively correlated with both ER (estrogen receptor) beta (*p* = 0.001) and PRA (progesterone receptor A) (*p* = 0.037), while cytoplasmic staining was negatively correlated with ER alpha (*p* = 0.036). No correlations were found in the case of TR beta-1.

To perform statistical analysis, two different scores were calculated. In the first place, TR staining was binarized (negative vs. positive) by using median nuclear and median cytoplasmic IRS cut offs, respectively (Figure 1E,F). Hence, a case was scored as positive if either nuclear or cytoplasmic TR stain was above the median of the respective location (Figure 1E,F). By applying this algorithm (which is independent of subcellular localization), TR beta was detected in 98 out of 152 cases (64.5%), while a TR beta-1 signal was found in 106 out of 151 cases (70.2%). Using this binarized score, the presence of TR beta (regardless of its subcellular localization) was positively associated with the high-grade serous subtype (*p* < 0.001), advanced FIGO stage (*p* = 0.003), high grade (*p* = 0.003), presence of lymph node metastasis (*p* = 0.018) and patient age higher than 55 years (*p* = 0.002). TR beta-1 (as quantified using the binarized score) was not associated with clinico-pathological parameters (Supplementary Table S1).

**Figure 2:**
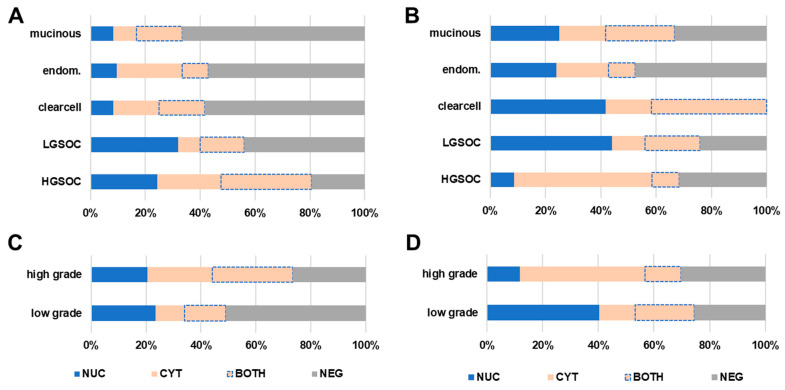
TR beta/TR beta-1 subcellular localization. The nuclear and cytoplasmic signal of TR beta and TR beta-1 were assessed independently by using a four-sided score. Relative fractions of each subcellular localization recognizing exclusive nuclear stain (NUC, blue), exclusive cytoplasmic stain (CYT, light orange), and the co-occurrence of nuclear and cytoplasmic signal (BOTH, light-orange with dotted blue line), as well as the complete loss of both (NEG, grey), were calculated. This four-sided score was correlated with histologic subtype and grade. The subcellular distribution of TR beta (**A**,**C**) and TR beta-1 (**B**,**D**) significantly changed with ovarian cancer histologic subtypes and grade (**C**,**D**).

In order to further investigate the subcellular distribution pattern, a four-sided score, recognizing exclusive nuclear stain (NUC), exclusive cytoplasmic stain (CYT), and the co-occurrence of nuclear and cytoplasmic signal (BOTH), as well as the complete loss of both (NEG), was calculated. By applying this four-sided score, an exclusively nuclear stain of TR beta was found in 21.1% (*n* = 32). Complete or incomplete shift of TR beta to the cytoplasm was detected in 43.4% (*n* = 66) of cases. Regarding the TR beta-1 isoform, cytoplasmic shift of the receptor was detected in 49.3% (*n* = 75) of cases. Solely nuclear stain only accounted for 31 (20.4%) cases investigated for TR beta-1. The subcellular localization of TR beta and beta-1 was tested for association with clinico-pathological parameters (Figure 2A–D and Supplementary Table S1). The subcellular distribution of TR beta/beta-1 was found to be significantly different when high- and low-grade serous cases were compared (TR beta: *p* = 0.028, TR beta-1: *p* < 0.001). The same applied for high- vs. low-grade cases compared across subtypes (TR beta: *p* = 0.010, TR beta-1: *p* < 0.001; Figure 2C,D and Supplementary Table S1). The subcellular distribution also changed with histologic subtype in general (Figure 2A,B and Supplementary Table S1). However, for more rare histologic subtypes, the number of cases per subgroup was too low to perform proper statistics. FIGO stage also influenced the subcellular localization of TR beta (*p* = 0.01) and TR beta-1 (*p* = 0.035) (Supplementary Table S1). Finally, for TR beta, localization changed with patient age (*p* = 0.004) (Supplementary Table S1).

### 2.3. TR Beta/Beta-1 and Ovarian Cancer Prognosis

**Figure 3:**
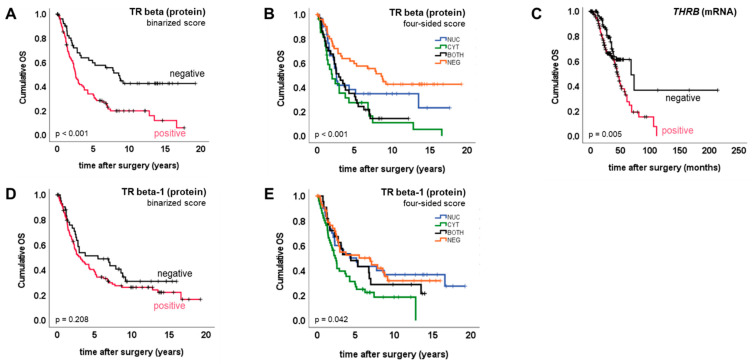
TR beta/TR beta-1 and overall survival. Kaplan–Meier plots (**A**–**E**) were drawn to illustrate overall survival of patient subgroups defined by TR IHC/mRNA.

TR beta, as well as its isoform TR beta-1, were tested for association with overall survival (OS). By applying the binarized score, we detected TR beta positivity to predict significantly reduced overall survival (median OS (pos. vs. neg.) 2.5 vs. 8.5 years, *p* < 0.001) (Figure 3A). TR beta-1 positivity was not prognostic for overall survival (Figure 3D). To verify the prognostic value of TR beta in an independent cohort, and by using an independent method, survival analysis was repeated by employing a publicly available gene expression dataset [13]. Like the TR beta protein, *THRB* gene expression (mRNA) on the publicly available cohort was significantly associated with reduced overall survival (median OS (pos. vs. neg.) 45 vs. 68 months, *p* = 0.005) (Figure 3C). This also remained significant in the sub-cohort of optimally debulked patients (*n* = 160, *p* = 0.04).

Multivariate analysis, taking into account FIGO stage, the presence of lymph node metastasis, grade and patient age, was performed to test whether TR beta, as detected by immunohistochemistry, might also be an independent prognostic factor. Though the presence of TR beta was prognostic in univariate analysis, as explained above, this was no longer significant within multivariate testing (*p* = 0.132; HR = 1.6) (Supplementary Table S2).

The four-sided score was used to test whether the subcellular localization pattern of TR beta or TR beta-1 might be associated with prognosis. Regarding both TR beta (*p* < 0.001) and TR beta-1 (*p* = 0.042), overall survival did significantly change depending on subcellular localization (Figure 3B,E). Complete or incomplete shift of TR beta to the cytoplasm was prognostic for shortened overall survival when compared with the remaining cases, respectively (complete: median OS (CYT vs. remaining) 2.0 vs. 3.8 years, *p* = 0.005; incomplete: median OS (CYT+BOTH. vs. NUC+NEG) 2.5 vs. 5.2 years, *p* < 0.001). A similar result was retrieved when the presence of TR beta in the cytoplasm was contrasted with the complete loss of the receptor (median OS (CYT+BOTH. vs. NEG) 2.5 vs. 8.5 years, *p* < 0.001). However, in multivariate testing, the shift of TR beta to the cytoplasm (CYT+BOTH) did not remain prognostic for shortened overall survival (*p* = 0.106; HR = 1.6) (Supplementary Table S2).

These observations were confirmed by repeating the analysis for TR beta-1. Again, cytoplasmic TR beta-1 (CYT+BOTH) turned out to be a negative prognosticator when compared with the remaining cases (NUC+NEG) (*p* = 0.018), or with those that showed complete loss (NEG) of TR beta-1 (*p* = 0.043). Cytoplasmic shift (CYT+BOTH) did not remain significant within multivariate testing (*p* = 0.143; HR = 1.6).

## 3. Discussion

A relevant fraction of samples showed not only nuclear, but also cytoplasmic, stain of TR beta and/or beta-1. Regarding TR beta-1, median cytoplasmic staining scores were even higher than the respective nuclear scores. Although TRs were already discovered in the 1980s, and although the influence of thyroid hormones on body homeostasis is undoubted, the subcellular distribution of TRs, and especially its biological meaning in cancer, has only recently come into research focus. Initially, TRs were identified as DNA binding molecules that act as (hetero-)dimers and facilitate the transcription of target genes [3,4]. Though TRs are supposed to primarily reside in the nucleus, TR alpha-1 and TR beta-1 have also been identified to quickly shuttle between nucleus and cytoplasm [14,15,16]. Translocation across the nuclear envelope is mediated by TRs interacting with importins and exportins, which recognize nuclear import and export signals on the TR protein [15]. The dynamic transport of TRs is, hence, seen as a major regulator of TR signaling activity, and adds complexity to thyroid hormone signaling activities. Therefore, the intracellular localization of TRs is more and more recognized as a major factor to consider in disease pathogenesis [17]. During the last few years, several extranuclear signaling activities of TRs have been discovered. For instance, TR beta has been found to interact with the p85 subunit of PI3-kinase, and to stimulate PI3K- and mTOR-mediated signaling [12,18]. However, most studies published on the in-situ analysis of TR in human cancer tissue do not exactly report on the subcellular distribution of TRs. Though not reporting on the exact distribution, a comprehensive study investigating almost 800 breast cancer patients found that TR beta-1 was predominantly localized in the cytoplasm of cancer cells [19]. A former study from our group also investigated TR beta-1 in breast cancer and found cytoplasmic expression in 43% of the cases [20]. This is similar to the finding reported here in ovarian cancer, i.e., the cytoplasmic localization of TR beta in 44% and TR beta-1 in 49% of tissue samples. Unfortunately, to the best of our knowledge, there is no study on TR beta/TR beta-1 distribution in ovarian cancer, to which our in-situ data could be compared.

TR beta turned out to be prognostic for shortened overall survival in univariate analysis. This was initially observed by contrasting TR beta positive vs. negative cases as determined by immunohistochemistry. To validate this finding, we used gene expression data stemming from a publicly available dataset [13]. When the expression of *THRB* (encoding TR beta) was tested for association with patients’ overall survival in an independent sample set, the IHC results of our cohort could be confirmed. These data are also supported by an analysis published on the TCGA cohort of endometrial cancer [21]. Applying the *receptLoss* algorithm, the authors highlighted that loss of *THRB* gene expression was linked to favorable prognosis in endometrial cancer [21]. In addition, TR beta mutations have been demonstrated to exert tumor-promoting activity in different types of cells [22]. Though the *THRB* mutational status of tissue samples studied in the current analysis is not known, human tumors in general have been reported to carry multiple TR mutations that might influence both the biologic and prognostic meaning of TR beta [23]. In addition, as our data presented above propose that especially cytoplasmic TR beta predicts shortened survival, another hypothesis seems to be reasonable. Since cytoplasmic TR beta was discovered to stimulate PI3K and mTOR signaling, two pathways highly relevant for tumor cell growth and survival, this ‘non-genomic’ activity of cytoplasmic TR beta might also explain why (cytoplasmic) TR beta was linked to ovarian cancer aggressiveness in our analysis [18,24]. Both TR beta positivity, as well as its cytoplasmic shift, displayed a correlation with the high-grade serous subtype, high grade per se and advanced FIGO stage. Therefore, it could be hypothesized that the prognostic value of TR beta, or its subcellular localization observed in the univariate analysis, might be due to correlation with other variables that influence prognosis. Hence, the prognostic significance of TR beta was lost in multivariate testing. Whether the correlation is caused solely by chance, or whether there is a tumor-biologic reason that links TR beta with parameters related to disease aggressiveness (e.g., advanced FIGO stage, high grade, histologic subtype), remains to be elucidated. Finally, the biologic meaning of TR beta and its prognostic role might also depend on the cancer entity studied, as several analyses performed in breast, thyroid and gastrointestinal cancers propose TR beta to predict favorable prognosis [19,20,25].

Since the nature of our analysis reported here is descriptive, data need to be interpreted with caution, and functional validation is warranted in order to draw more definite conclusions. This becomes particularly obvious as, due to the retrospective style of the current study, important clinical information such as surgical resection status is not available, and treatment options have changed since the tumor tissue samples were collected. Thus, to consider these aspects at least partly, survival analysis was repeated in a more up-to-date, publicly available dataset, which was also curated for debulking status.

Taken together, this study reported TR beta and beta-1 to be widely expressed in ovarian cancer. In addition, both TRs were, by far, not only expressed in the tumor cell nuclei, but were commonly found in the cytoplasm. Especially those cases that showed complete or incomplete cytoplasmic shift of TR beta had significantly shortened overall survival. The prognostic significance of TR beta or its localization was lost in multivariate testing.

## 4. Materials and Methods

### 4.1. Tissue Samples

Ovarian cancer tissue samples were collected from 153 patients that had undergone tumor debulking surgery from 1990 to 2002 at the Department of Obstetrics and Gynecology of the Ludwig-Maximilians-University Munich. Histopathological evaluation was performed according to the criteria of the International Federation of Gynecologists and Obstetricians (FIGO) and the World Health Organization (WHO) by experienced gynecologic pathologists. When high- and low-grade cases were compared across histologic subtypes, high grade was defined according to the WHO definition: high-grade serous, clear cell (no grading), poorly differentiated (G3) endometroid and poorly differentiated (G3) mucinous cases. Remaining subtypes and grades were defined as low grade. Cases diagnosed for ovarian tumors of low malignant potential were excluded from the study. Patient charts, aftercare files and the Munich tumor registry database were used to perform clinical annotation of the tissue samples.

### 4.2. Immunohistochemistry

The general immunohistochemistry procedure of TR beta and TR beta-1 detection on FFPE sections was published by our group before [26,27]. For the current study, TR beta, as well as TR beta-1 protein, was stained using rabbit polyclonal antibodies obtained from Upstate Cell signaling solutions (Upstate Cell Signaling Solutions (Lake Placid, NY, USA)) and Zytomed (Berlin, Germany), respectively. Staining was performed by applying the ZytoChem Plus HRP Polymer System kit (Zytomed). In order to control for unspecified signals, appropriate positive and negative controls were included in each experiment. Pre-immune IgG, instead of the primary antibodies, served as negative controls.

Two independent observers quantified the staining signals by applying a well-established scoring system (IR-score) by consensus. To calculate the IR-score, optical staining intensity (graded as 0: no, 1: weak, 2: moderate and 3: strong staining) and the percentage of stained cells (0: no staining, 1: ≤10% of the cells, 2: 11–50% of the cells, 3: 51–80% of the cells and 4: ≥81% of the cells) are multiplied, resulting in a semi-quantitative score ranging from 0 to 12. Numerous studies published by our group already used this scoring method in the past [28,29,30]. Median nuclear and cytoplasmic TR beta/beta-1 expression was taken to discriminate negative vs. positive cases, respectively.

### 4.3. Statistical Analysis Methods

The IBM statistic package SPSS (version 28, IBM, Armonk, NY, USA) and Microsoft Excel (v 2201, Microsoft, Redmond, WA, USA) were used to perform statistical analysis and to plot graphs, respectively. Clinico-pathological parameters and TR beta/beta-1 status were tested for independence by chi-square test. The Spearman correlation coefficient was used for correlation analysis. Differences between IR-scores were tested using the Mann–Whitney U test. Survival times of positive vs. negative cases were compared by applying the Kaplan–Meier method, and differences in patient overall survival were tested for significance by using the chi-square statistics of the log-rank test. The prognostic role of the subcellular distribution of TR beta/beta-1 was tested accordingly.

To validate our results, we questioned whether THRB remains prognostic for OS when detected by an independent method and on an independent cohort. Therefore, data from a publicly available dataset (GSE9891), initially published by Tothill et al., were investigated [13]. Tothill et al. used the Affymetrix (Affymetrix, Santa Clara, CA, USA) Human Genome U133 Plus 2.0 Array, which quantifies expression of virtually all known human genes, and made the data publicly available on Gene Expression Omnibus (GEO) [13]. To back up our results, gene expression (mRNA) data of *THRB* deriving from the study of Tothill et al. were analyzed by using the KM plotter [31]. Gene expression of *THRB,* as detected by the Affymetrix probe set 229657_at, was used for the calculations. Data were assumed to be statistically different in case of *p* < 0.05.

## Figures and Tables

**Table 1:** Patients’ characteristics.

		*n* (Total)	*n*	%
**Histology**		153		
	HGSOC		82	54
	LGSOC		26	17
	clear cell		12	8
	endometrioid		21	14
	mucinous		12	8
**FIGO stage**		152		
	I, II		43	28
	III, IV		109	72
**pN**		93		
	N0		41	45
	N+		51	55
**Patient age**		152		
	≤55 years		62	41
	>55 years		90	59

Abbreviations: HGSOC—high-grade serous ovarian cancer, LGSOC—low-grade serous ovarian cancer, pN—pathological lymph node status (N0: no lymph node metastasis detected, N+: lymph node metastasis).

## Data Availability

IHC data are available from the corresponding author on reasonable request. Gene expression data were from GSE9891 (https://www.ncbi.nlm.nih.gov/geo/query/acc.cgi?acc=GSE9891) and analyzed by using Kaplan-Meier plotter (https://kmplot.com/analysis/index.php?p=service&cancer=ovar) [13,31].

## Supplementary Materials

The following are available online at www.mdpi.com/article/10.3390/ijms23052698/s1.
