# Supplementary material for: Subcellular Distribution of Thyroid Hormone Receptor Beta in Ovarian Cancer
Source: Int J Mol Sci. 2022 Feb 28;23(5):2698. doi: 10.3390/ijms23052698 (PMC8910424; doi:10.3390/ijms23052698)
Supplement: Supplementary file 1 [file ijms-23-02698-s001.zip › ijms-1571472-supplementary.pdf]

International Journal of
Molecular Sciences

MDPI

Research Article

Subcellular Distribution of Thyroid Hormone Receptor Beta in Ovarian Cancer

Sabine Heublein[1,2*], Udo Jeschke[2,3], Cornelia Sattler[1,4], Christina Kuhn[2,3], Anna Hester[2], Bastian Czogalla[2], Fabian Trillsch[2], Sven Mahner[2], Doris Mayr[5], Elisa Schmoeckel[5], Nina Ditsch[2,3]

Supplementary Tables:

Table S1. Abbreviations: pN - pathological lymph node status (N0: no lymph node metastasis detected, N+: lymph node metastasis), y - year.

		TR beta			TR beta-1			TR beta					TR beta-1				
		**negative**	**positive**	**p**	**negative**	**positive**	**p**	**NUC**	**CYT**	**BOTH**	**NEG**	**p**	**NUC**	**CYT**	**BOTH**	**NEG**	**p**
**Histology**	**serous**	27	80	<.001	31	75	ns	28	21	31	27	<.001	18	44	13	32	0.024
	**other**	27	18		14	31		4	8	6	27		13	8	10	14	
**FIGO**	**I, II**	23	20	0.003	10	32	ns	9	3	8	23	0.01	14	10	8	10	0.035
	**III, IV**	30	78		35	73		23	26	29	30		16	42	15	36	
**grade**	**low**	24	23	0.003	12	35	ns	11	5	7	24	0.01	19	6	10	12	<.001
	**high**	27	75		30	71		21	24	30	27		12	46	13	31	
**N**	**N0**	22	19	0.018	13	26	ns	7	5	7	22	ns	10	11	5	14	ns
	**N+**	15	36		14	37		12	10	14	15		9	18	10	14	
**patient age**	**≤ 55 y**	31	31	0.002	18	43	ns	14	6	11	31	0.004	14	17	12	18	ns
	**> 55 y**	23	66		27	62		17	23	26	23		16	35	11	28	

Table S2. * High grade was defined according to the WHO definition: high grade serous, clear cell (no grading), poorly differentiated (G3) endometroid and poorly differentiated (G3) mucinous cases. Remaining subtypes and grades were defined as low grade.

	**HR**	**Sig.**	**95,0% CI**		**HR**	**Sig.**	**95,0% CI**	
			**Lower**	**Upper**			**Lower**	**Upper**
FIGO (I, II vs. III, IV)	**0.44**	**0.30**	**0.09**	2.08	0.55	0.45	0.12	2.57
grade* (low vs. high)	2.84	**0.003**	1.42	5.68	2.90	**0.002**	1.46	5.78
pN (negative vs. positive)	6.66	**0.009**	1.60	27.76	5.63	**0.019**	1.33	23.73
patient age (≤ 55 y vs. > 55 y)	0.74	0.31	0.41	1.32	0.73	0.29	0.41	1.31
TR beta (negative vs. positive)	1.64	0.13	0.86	3.12	na	na	na	na
TR beta (remaining vs. CYT+BOTH)	na	na	na	na	1.61	0.11	0.90	2.87
